# Pulmonary fibrosis associated with psychotropic drug therapy: a case report

**DOI:** 10.1186/1752-1947-3-126

**Published:** 2009-11-16

**Authors:** Clare Thornton, Toby M Maher, David Hansell, Andrew G Nicholson, Athol U Wells

**Affiliations:** 1 Interstitial lung disease Unit, Royal Brompton Hospital, Sydney Street, SW3 6NP, UK; 2 Department of Radiology, Royal Brompton Hospital, Sydney Street, SW3 6NP, UK; 3 Department of Pathology, Royal Brompton Hospital, Sydney Street, SW3 6NP, UK

## Abstract

**Introduction:**

Sertraline and Risperidone are commonly used psychotropic drugs. Sertraline has previously been associated with eosinopilic pneumonia. Neither drug is recognised as a cause of diffuse fibrotic lung disease. Our report represents the first such case.

**Case Presentation:**

We describe the case of a 33 year old Asian male with chronic schizophrenia who had been treated for three years with sertraline and risperidone. He presented to hospital in respiratory failure following a six month history of progressive breathlessness. High resolution CT scan demonstrated diffuse pulmonary fibrosis admixed with patchy areas of consolidation. Because the aetiology of this man's diffuse parenchymal lung disease remained unclear a surgical lung biopsy was undertaken. Histological assessment disclosed widespread fibrosis with marked eosinophillic infiltration and associated organising pneumonia - features all highly suggestive of drug induced lung disease. Following withdrawal of both sertraline and risperidone and initiation of corticosteroid therapy the patient's respiratory failure resolved and three years later he remains well albeit limited by breathlessness on heavy exertion.

**Conclusion:**

Drug induced lung disease can be rapidly progressive and if drug exposure continues may result in respiratory failure and death. Prompt recognition is critical as drug withdrawal may result in marked resolution of disease. This case highlights sertraline and risperidone as drugs that may, in susceptible individuals, cause diffuse pulmonary fibrosis.

## Introduction

Prescribed medications are an important cause of diffuse pulmonary fibrosis. Over 300 separate drugs having been associated with fibrotic lung disease[1]. Because patients with pulmonary fibrosis frequently present late in the course of their illness and with advanced disease, a diagnosis of drug induced fibrosis is often difficult to confirm. In many cases of drug induced lung disease the progression of fibrosis can be halted by withdrawal of the causative agent. It is crucial therefore that drugs are considered as a cause of fibrosis especially in cases exhibiting atypical features.

The selective seretonin reuptake inhibitor sertraline and the atypical neuroleptic risperidone are commonly used psychotropic drugs that are sometimes used in combination for the treatment of chronic schizophrenia. Sertraline has been reported in two patients as being the cause of eosinophilic pneumonia[2,3]. Barnes *et al *describe a 40 year old lady who had been taking sertraline in combination with clomipramine and a benzodiazepine for one week[2]. She presented acutely with diffuse pulmonary infiltrates and a peripheral blood eosinophillia. Her condition resolved following withdrawal of sertraline. In the second reported case a 34 year old lady treated with sertraline, at an initial dose of 100 mg daily for three months increasing to 200 mg daily for a further month presented acutely in respiratory failure. Chest X-ray demonstrated diffuse alveolar infiltrates and an eosinophillia was found on bronchoalveolar lavage[3]. A transbronchial biopsy in this case was consistent with eosinophilic pneumonia. The patient recovered fully following withdrawal of sertraline and treatment with oral corticosteroids.

In this report we describe the case of a 33 year old man with chronic schizophrenia treated with risperidone and sertraline who subsequently developed pulmonary fibrosis. He presented late in the course of his disease in severe respiratory failure. Despite this he has subsequently responded well to withdrawal of his psychotropic medication. We believe that in this case the clinical history, high resolution CT findings, surgical lung biopsy and subsequent clinical course all strongly support a diagnosis of sertraline induced pulmonary fibrosis. Neither sertraline nor risperidone have previously been described in the literature as a cause of pulmonary fibrosis.

## Case Presentation

A 33 year old Asian male with chronic schizophrenia was admitted to hospital with a six month history of inexorably progressive dyspnoea associated with a dry cough. At presentation he was in severe respiratory failure (Arterial blood gas on room air PaO2 7.74 kPa, PaCO2 4.17 kPa). He had been receiving treatment with risperidone 2 mg daily and sertraline 50 mg daily for 3 years. He was a current tobacco smoker with a five pack year smoking history. He gave no history of recent travel, illicit substance abuse or exposure to known pneumotoxic substances.

**Figure 1:**
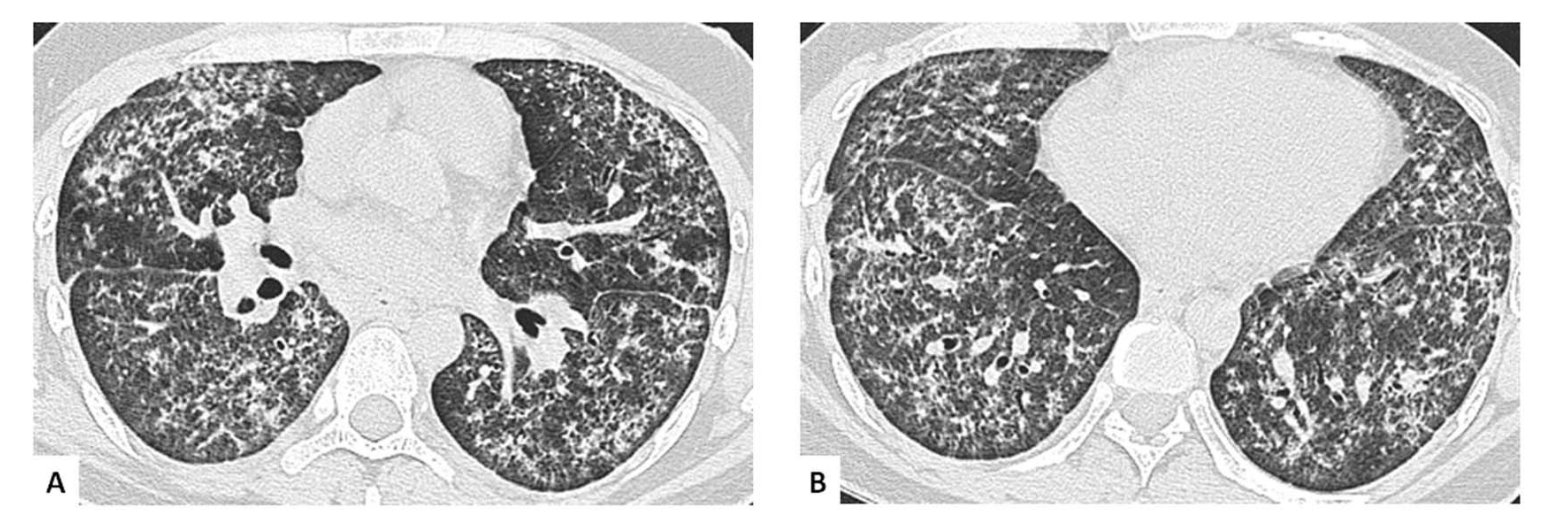
**High resolution thoracic CT at time of diagnosis**. High resolution CT sections at the level of a) the hila and b) the lung bases demonstrate widespread, bilateral ground glass change, multifocal patches of consolidation and evidence of fibrosis with fine reticulation and traction bronchiectasis.

**Figure 2:**
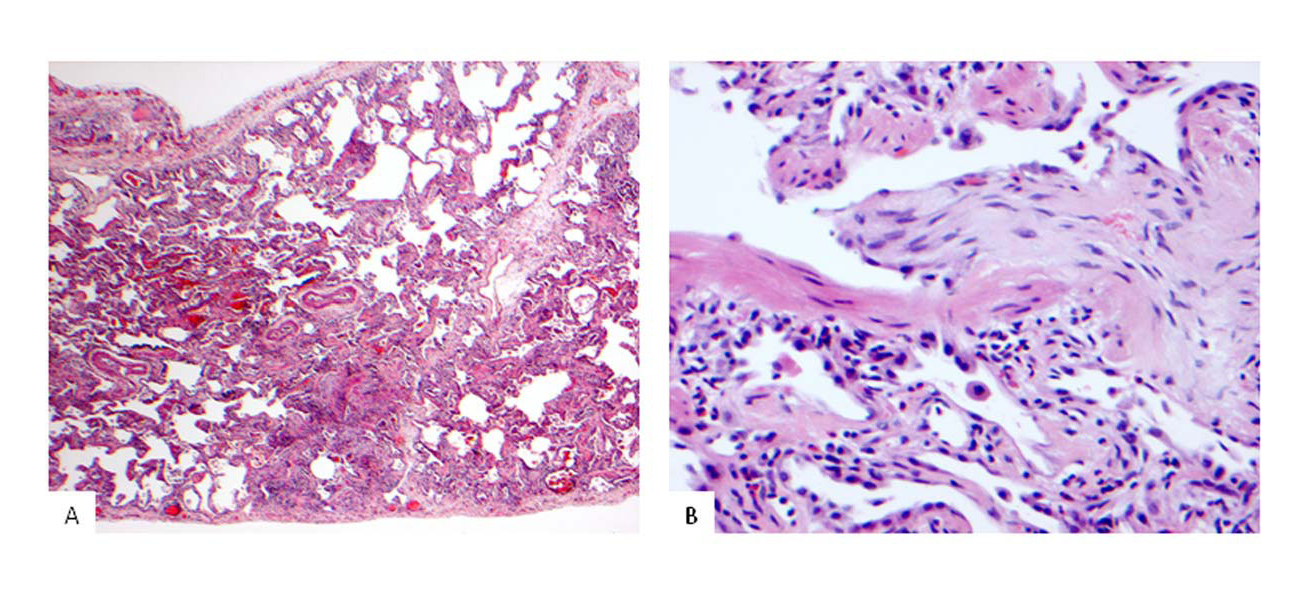
**Photomicrographs of left sided surgical lung biopsy**. 2a) The lung shows a histological pattern of fibrotic NSIP with diffuse established interstitial fibrosis associated with moderate chronic inflammation (×20 magnification). 2b) The lung at high power, shows focal organising pneumonia within a respiratory bronchiole with abundant eosinophils in the adjacent interstitium.

Physical examination disclosed fine bibasal crackles and finger clubbing. ESR was elevated at 43 mm/h. Rheumatoid factor was 1 in 40. Anti-nuclear antibodies, ANCA, double stranded DNA antibodies and extractable nuclear antigens were all negative. High resolution CT demonstrated diffuse ground glass attenuation with marked reticular change most prominent in the lower lobes (Figure 1). Also present were multiple discrete areas of consolidation. Bronchoscopy was unremarkable. Broncho-alveolar lavage revealed 26% neutrophils (normal < 7%) and 7.7% eosinophils (normal < 3%). Bronchial washings were negative on microscopy and culture for bacteria, fungi and mycobacteria. Because of uncertainty over the diagnosis a surgical lung biopsy was undertaken. Left upper and lower lobe surgical lung biopsies showed a pattern predominantly of fibrotic non-specific interstitial pneumonia (NSIP), with coexistent eosinophilic infiltration and occasional foci of organizing pneumonia (Figure 2a and 2b).

**Figure 3:**
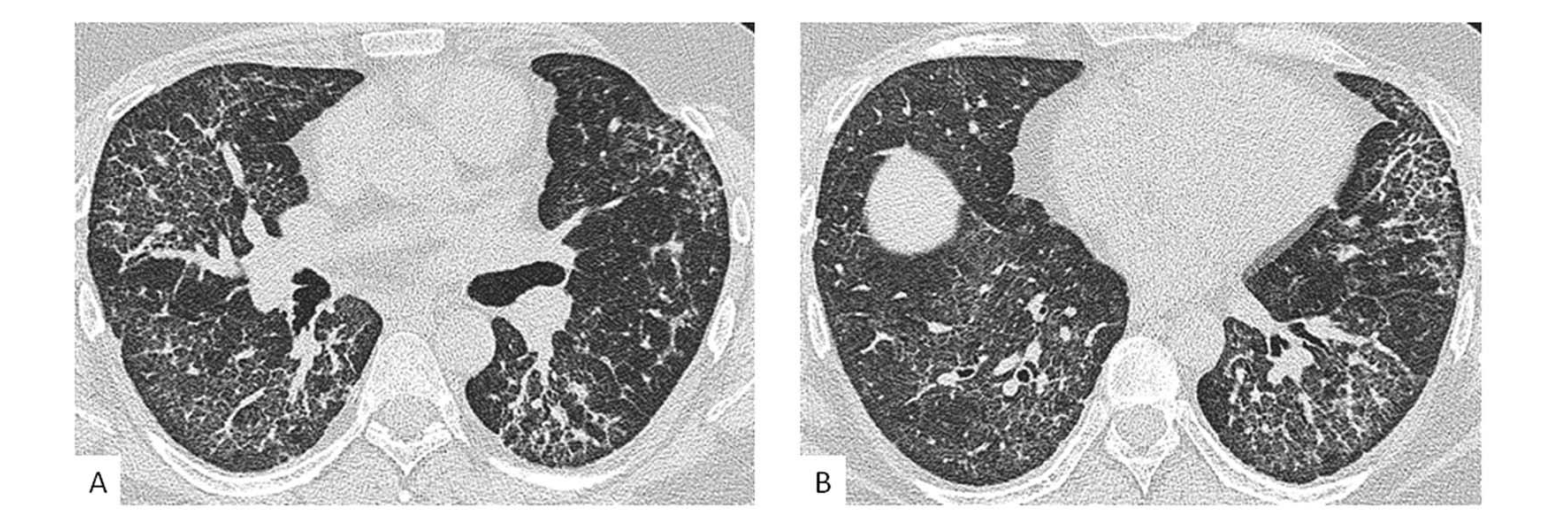
**High resolution thoracic CT three years after diagnosis**. High resolution CT sections at the level of a) the hilae and b) the lung bases demonstrate resolution of the previously noted consolidation and a reduction in ground glass attenuation. However evidence of fibrosis persists with bilateral reticular change and traction bronchiectasis. The dome of the right hemidiaphragm is visible in the image of the lung bases (b).

The combination of rapid clinical progression, unusual CT appearances, eosinophilia on broncho-alveolar lavage, biopsy showing prominent eosinophilic infiltration in association with fibrotic NSIP and an absence of alternative aetiologies strongly favoured a diagnosis of drug induced pulmonary fibrosis. Consequently sertraline and risperidone were stopped. On the advice of the patient's usual psychiatric team haloperidol was introduced as replacement anti-psychotic therapy. For the pulmonary fibrosis, treatment was commenced with intravenous methylprednisolone 1 g once weekly and cyclophosphamide 600 mg/m^2 ^every three weeks for a total of six doses. This was then converted to low dose oral prednisolone and azathioprine 150 mg daily with the prednisolone slowly being weaned over the succeeding twelve months. Initial recovery was complicated by the development of a left sided pneumothorax. However four weeks after withdrawal of the antipsychotic medication the patient was ambulatory and no longer in respiratory failure. Three years on from diagnosis our patient is clinically stable on treatment with azathioprine alone. HRCT continues to demonstrate evidence of widespread interstitial fibrosis but other changes, including the ground glass attenuation and patchy consolidation, have resolved (Figure 3).

## Discussion

Diagnosing drug induced lung disease is always challenging[1]. Patients with pulmonary fibrosis typically present at a late stage in their disease making temporal associations of disease with the commencement of prescribed medication difficult. Furthermore, as in our case, the severity of lung damage seen in patients with drug induced pulmonary fibrosis rarely makes it appropriate to re-challenge patients with the suspected causative agent. Clinicians managing suspected drug induced lung disease are therefore left to exclude other potential causes of fibrosis before relying on atypical or ancillary features of a case to establish a diagnosis.

Potential differential diagnoses for our patient at the time of his initial presentation included occult connective tissue disease or idiopathic NSIP. The absence of auto-antibodies and the subsequent failure to manifest extra-thoracic symptoms go strongly against the possibility of connective tissue disease associated NSIP. The initial CT appearance and histology and the subsequent clinical course are not in keeping with a diagnosis of idiopathic NSIP [4]. We believe that the clinical, histological and radiological features of this case strongly favour a diagnosis of sertraline induced pulmonary fibrosis. This is borne out by the clinical and radiological response seen following drug withdrawal. Areas of consolidation and ground glass attenuation on CT (areas that likely correspond to organising pneumonia and eosinophillic inflammation on biopsy) resolved. Furthermore, pronounced broncho-alveolar lavage eosinophilia and the marked infiltration of eosinophils into areas of fibrosis on biopsy are both frequent findings in drug induced lung disease[5]. The severity of our patient's disease at presentation was such that we felt it necessary to commence therapy with intravenous methylprednisolone and cyclophosphamide. It is therefore conceivable that his improvement was due to our therapeutic intervention and unrelated to the discontinuation of his medication. Against this however is the subsequent three year stability in this man's disease despite tapering of corticosteroid and immunosuppressant dosages. Such stability, even following treatment, is unusual in idiopathic fibrosing lung conditions.

We believe that in our patient's case, sertraline was the likeliest cause of his fibrosis. Neither sertraline nor risperidone have previously been described as causing pulmonary fibrosis. Sertraline however, is a described cause of eosinophilic pneumonia and furthermore other drugs within the class of selective serotonin reuptake inhibitors have been associated with pulmonary fibrosis, granulomatous lung disease and hypersensitivity pneumonitis and acute lung injury [6-8]. Risperidone and the atypical neuropleptics have not been reported to have pulmonary side effects. Furthermore, our patient was continued on haloperidol, a drug that shares many of the pharmacodynamic features of the atypical neuroleptics including risperidone [9], without this causing further progression of his respiratory disease. So, although it is impossible to rule out a role for risperidone in the development of fibrosis in our patient, sertraline would seem to be by far the likeliest causative agent.

In contrast to our case, in the two previously reported cases of eosinophilic pneumonia occurring in association with sertraline the affected individuals had only recently been started on the drug. The duration of our patient's therapy coupled with the chronicity of his symptoms and his late presentation may explain why histologically the fibrotic NSIP appeared to have evolved from a picture resembling eosinophilic pneumonia. It is interesting to note that regions of eosinophilic pneumonia, as judged by HRCT, resolved following discontinuation of sertraline and risperidone. Established fibrotic NSIP, although not progressing, did not resolve with therapy or discontinuation of sertraline and risperidone. The pathogenesis of fibrotic lung diseases including drug induced pulmonary fibrosis remains poorly understood. In our patient it seems likely that chronic eosinophilic infiltration has resulted in persistent airway epithelial injury and through this process has induced an aberrant, fibrogenic wound healing response in a manner akin to that postulated to underlie the pathogenesis of idiopathic pulmonary fibrosis[10]. A similar progression of eosinophilic pneumonia to chronic fibrosis has previously been reported by Yoshida *et al *in a patient with idiopathic disease[11]. A further possible precipitant for our patient progressing to fibrosis is the fact that he was a smoker. Smoking appears to be a potential co-factor in the development of a number of fibrotic lung conditions[12].

## Conclusion

This case highlights the need for physicians to be alert to the possibility that sertraline and possibly risperidone may be a potential cause of eosinophilic pneumonia and progressive pulmonary fibrosis. It is important that drugs are recognized as a cause of fibrotic lung disease because, as in the case presented in this report, prompt early treatment and drug cessation can arrest disease progression and lead to a marked improvement in respiratory function.

## Abbreviations

ANCA: Anti-neutrophil cytoplasmic antibodies; ESR: Erythrocyte sedimentation rate; HRCT: High resolution computerised tomography; NSIP: non-specific interstitial pneumonia.

## Consent

Written informed consent was obtained from the patient for publication of this case report and accompanying images. A copy of the written consent is available for review by the Editor-in-Chief of this journal.

## Competing interests

The authors declare that they have no competing interests.

## Authors' contributions

CT and TM were major contributors to the writing of the manuscript. DH analyzed and interpreted the radiological data and reviewed the manuscript. AN analyzed and interpreted the histology and contributed to the writing of the manuscript. AW analyzed and interpreted the clinical data and contributed to the writing of the manuscript. All authors have read and approved the final manuscript.

## References

[B1] Camus P Fanton A Bonniaud P Camus C Foucher P Interstitial lung disease induced by drugs and radiation Respiration 2004 71 301 326 10.1159/000079633 15316202

[B2] Barnes MT Bascunana J Garcia B varez-Sala JL Acute eosinophilic pneumonia associated with antidepressant agents Pharm World Sci 1999 21 241 242 10.1023/A:1008727421475 10550851

[B3] Haro M Rubio M Xifre B Castro P [Acute eosinophilic pneumonia associated to sertraline] Med Clin (Barc) 2002 119 637 638 12433344 10.1016/s0025-7753(02)73522-7

[B4] Travis WD Hunninghake G King TE Jr Lynch DA Colby TV Galvin JR Idiopathic nonspecific interstitial pneumonia: report of an American Thoracic Society project Am J Respir Crit Care Med 2008 177 1338 1347 10.1164/rccm.200611-1685OC 18388353

[B5] Costabel U Uzaslan E Guzman J Bronchoalveolar lavage in drug-induced lung disease Clin Chest Med 2004 25 25 35 10.1016/S0272-5231(03)00143-6 15062594

[B6] Gonzalez-Rothi RJ Zander DS Ros PR Fluoxetine hydrochloride (Prozac)-induced pulmonary disease Chest 1995 107 1763 1765 10.1378/chest.107.6.1763 7781383

[B7] Vandezande LM Lamblin C Wallaert B [Interstitial lung disease linked to fluoxetine] Rev Mal Respir 1997 14 327 329 9411618

[B8] de Kerviler E Tredaniel J Revlon G Groussard O Zalcman G Ortoli JM Fluoxetin-induced pulmonary granulomatosis Eur Respir J 1996 9 615 617 10.1183/09031936.96.09030615 8730028

[B9] Pajonk FG Risperidone in acute and long-term therapy of schizophrenia--a clinical profile Prog Neuropsychopharmacol Biol Psychiatry 2004 28 15 23 10.1016/S0278-5846(03)00164-7 14687852

[B10] Maher TM Wells AU Laurent GJ Idiopathic pulmonary fibrosis: multiple causes and multiple mechanisms? Eur Respir J 2007 30 835 839 10.1183/09031936.00069307 17978154

[B11] Yoshida K Shijubo N Koba H Mori Y Satoh M Morikawa T Chronic eosinophilic pneumonia progressing to lung fibrosis Eur Respir J 1994 7 1541 1544 10.1183/09031936.94.07081541 7957844

[B12] Wells AU Nicholson AG Hansell DM Challenges in pulmonary fibrosis. 4: smoking-induced diffuse interstitial lung diseases Thorax 2007 62 904 910 10.1136/thx.2004.031021 17909189 PMC2094243

